# Supplementary material for: Nonparametric Analysis of Thermal Proteome Profiles Reveals Novel Drug-binding Proteins
Source: Mol Cell Proteomics. 2019 Oct 3;18(12):2506–15. doi: 10.1074/mcp.TIR119.001481 (PMC6885700; doi:10.1074/mcp.TIR119.001481)
Supplement: supplemental Figs. S6 and S7 [file TIR119.001481_index.html]

Supplement to Non-parametric analysis of thermal proteome profiles reveals novel drug-binding proteins | Molecular & Cellular Proteomics

## Supplemental Data

- Supplementary Methods - Detailed description of the fitting procedures for the scaling parameter, melting points, and mean functions of the model.
- Table S1 - Spreadsheet containing the results of the NPARC approach and of the Tm-based approach for all datasets listed in Table 1.
- Supplementary Figures S1-S5 - PDF containing Supplementary Figures S1-S5.
- Supplementary Figure S6 -

  All proteins detected by the NPARC approach with Benjamini-Hochberg adjusted F-test p-values <= 0.01 in the staurosporine data.
- Supplementary Figure S7 -

  All proteins detected by the NPARC approach with Benjamini-Hochberg adjusted F-test p-values <= 0.01 in the ATP data.
