# Supplementary material for: Nonparametric Analysis of Thermal Proteome Profiles Reveals Novel Drug-binding Proteins
Source: Mol Cell Proteomics. 2019 Oct 3;18(12):2506–15. doi: 10.1074/mcp.TIR119.001481 (PMC6885700; doi:10.1074/mcp.TIR119.001481)
Supplement: Supplementary Methods [file 144658_1_supp_388240_px6jjs.pdf]

Supplementary information: Non-parametric analysis of thermal proteome profiles reveals novel drug-binding proteins

Dorothee Childs, Karsten Bach, Holger Franken, Simon Anders, Nils Kurzawa, Marcus Bantscheff, Mikhail Savitski and Wolfgang Huber

September 1, 2019

Estimating the scaling parameter σ_0^2

The scaled χ^2-distribution in Eqn. (5) of the main text can be alternatively expressed as a Γ-distribution with shape parameter

$$\alpha = \frac{d_1}{2} \tag{1}$$

and scale parameter

$$\theta = 2\sigma_0^2. \tag{2}$$

The shape and scale parameters are related to the mean M and variance V of the Γ-distribution as follows:

$$M = \alpha \cdot \theta \tag{3}$$
$$V = \alpha \cdot \theta^2. \tag{4}$$

Substituting (1) and (2) into (3) and (4) yields expressions for M and V in terms of d_1 and σ_0:

$$M = \sigma_0^2 \cdot d_1 \tag{5}$$
$$V = 2 \cdot \sigma_0^4 \cdot d_1. \tag{6}$$

Solving (5) and (6) for d_1 yields

$$d_1 = 2 \cdot \frac{M^2}{V}. \tag{7}$$

and for the scaling factor σ_0^2:

$$\sigma_0^2 = \frac{\theta}{2} = \frac{M}{2\alpha} = \frac{M}{d_1} = \frac{M}{2 \cdot \frac{M^2}{V}} = \frac{1}{2}\frac{V}{M}. \tag{8}$$

These equations coincide with those used by Brown's method [1], which proposes an adaptation of Fisher's method for combining multiple p-values to the scenario

of correlated tests by estimating χ^2-distribution parameters from the data in a similar manner. To increase robustness, we estimated M and V by the median and median absolute deviation (also called *D-estimates* by [2]) of the observed values of $RSS_1 - RSS_0$.

Model fitting

All mean functions were fitted by nonlinear least squares regression using the `nls` function in `R`. For the NPARC analysis, the melting curve model (Eqn. (3) in the main text) was fitted separately per protein to obtain $\mu(t)$, or per protein and treatment condition to obtain $\mu_c(t)$. To reproduce the results of the T_m-based approach (Figure 5), the model fits were repeated per replicate and treatment condition for each protein.

Summary of the T_m-based approach

The results of the T_m-based approach were obtained with the `R` package `TPP` [3]. This package, and the method it implements, are described in [4], and we only briefly summarize here. For each curve obtained by the replicate- and condition-wise model fits, T_m was calculated as $T_\mathrm{m} = a/(b - \ln(\frac{1-f_\infty}{0.5-f_\infty} - 1))$ so that it fulfilled $f(T_\mathrm{m}) = 0.5$. Before hypothesis testing on this parameter, *a priori* filters were applied to remove curves with undesirable shape or goodness-of-fit by setting a threshold on the R^2, the slope and the plateau parameters (Table 3). Within each replicate, the difference in T_m between the treatment and control conditions (ΔT_m) was computed per protein and converted to z-scores. Robust versions of the z-scores were computed by replacing the mean and standard deviation by quantiles of the empirical distributions of ΔT_m, namely the mean by the 0.5 quantile, and the standard deviation by the 0.8413 quantile for positive values, or the 0.1587 quantile for negative values. These quantities correspond to mean and standard deviation in the case of a normal distribution. In order to reduce the influence of values with high estimation uncertainty when calculating these quantiles, proteins were binned by the slopes of their curves, and z-scores were calculated separately for each bin as described in [5]. For each protein, p-values were calculated by comparing the z-scores to the normal distribution. To reach the final decision for each protein, the p-values were combined heuristically across replicates using the decision ruleset of Table 5.

References

1. Brown, M.B.: 400: A method for combining non-independent, one-sided tests of significance. Biometrics **31**, 987–92 (1975)
2. Marazzi, A.: Bootstrap tests for robust means of asymmetric distributions with unequal shapes. Computational Statistics & Data Analysis **39**, 503–28 (2002)
3. Childs, D., Kurzawa, N., Franken, H., Doce, C., Savitski, M., Huber, W.: TPP: Analyze Thermal Proteome Profiling (TPP) Experiments. (2019). R package version 3.12.0
4. Franken, H., Mathieson, T., Childs, D., Sweetman, G.M., Werner, T., Tögel, I., Doce, C., Gade, S., Bantscheff, M., Drewes, G., Reinhard, F.B.M., Huber, W., Savitski, M.M.: Thermal proteome profiling for unbiased identification of direct and indirect drug targets using multiplexed quantitative mass spectrometry. Nature Protocols **10**, 1567–93 (2015)
5. Cox, J., Mann, M.: MaxQuant enables high peptide identification rates, individualized p.p.b.-range mass accuracies and proteome-wide protein quantification. Nature Biotechnology **26**, 1367–72 (2008)
