# Supplementary material for: Nonparametric Analysis of Thermal Proteome Profiles Reveals Novel Drug-binding Proteins
Source: Mol Cell Proteomics. 2019 Oct 3;18(12):2506–15. doi: 10.1074/mcp.TIR119.001481 (PMC6885700; doi:10.1074/mcp.TIR119.001481)
Supplement: Supplementary Figure S6 [file 144658_1_supp_388214_px6cr4.pdf]

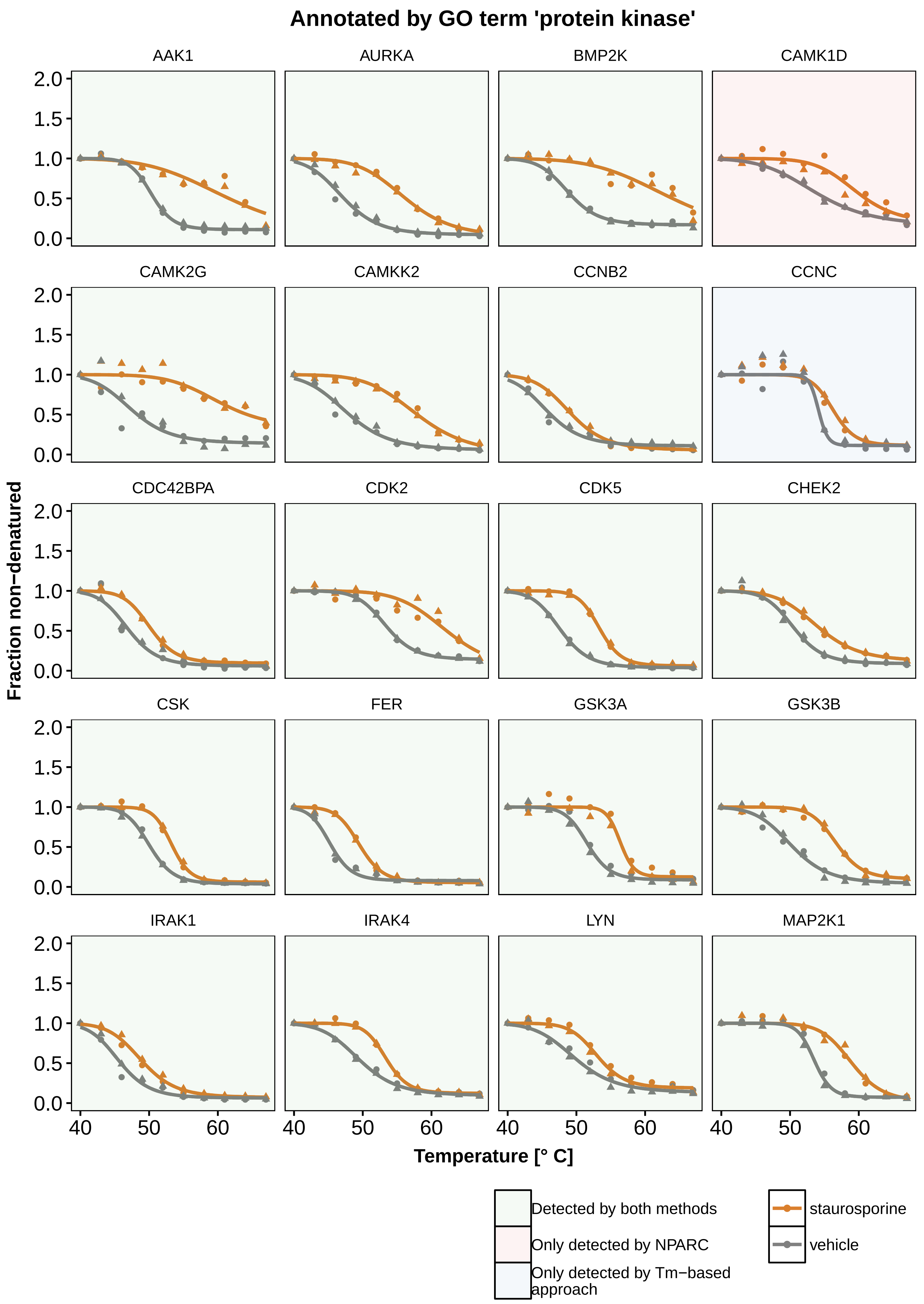

Annotated by GO term 'protein kinase'
AAK1
AURKA
BMP2K
CAMK1D
CAMK2G
CAMKK2
CCNB2
CCNC
CDC42BPA
CDK2
CDK5
CHEK2
CSK
FER
GSK3A
GSK3B
IRAK1
IRAK4
LYN
MAP2K1
Fraction non−denatured
0.0
0.5
1.0
1.5
2.0
40
50
60
Temperature [° C]
Detected by both methods
Only detected by NPARC
Only detected by Tm−based approach
staurosporine
vehicle

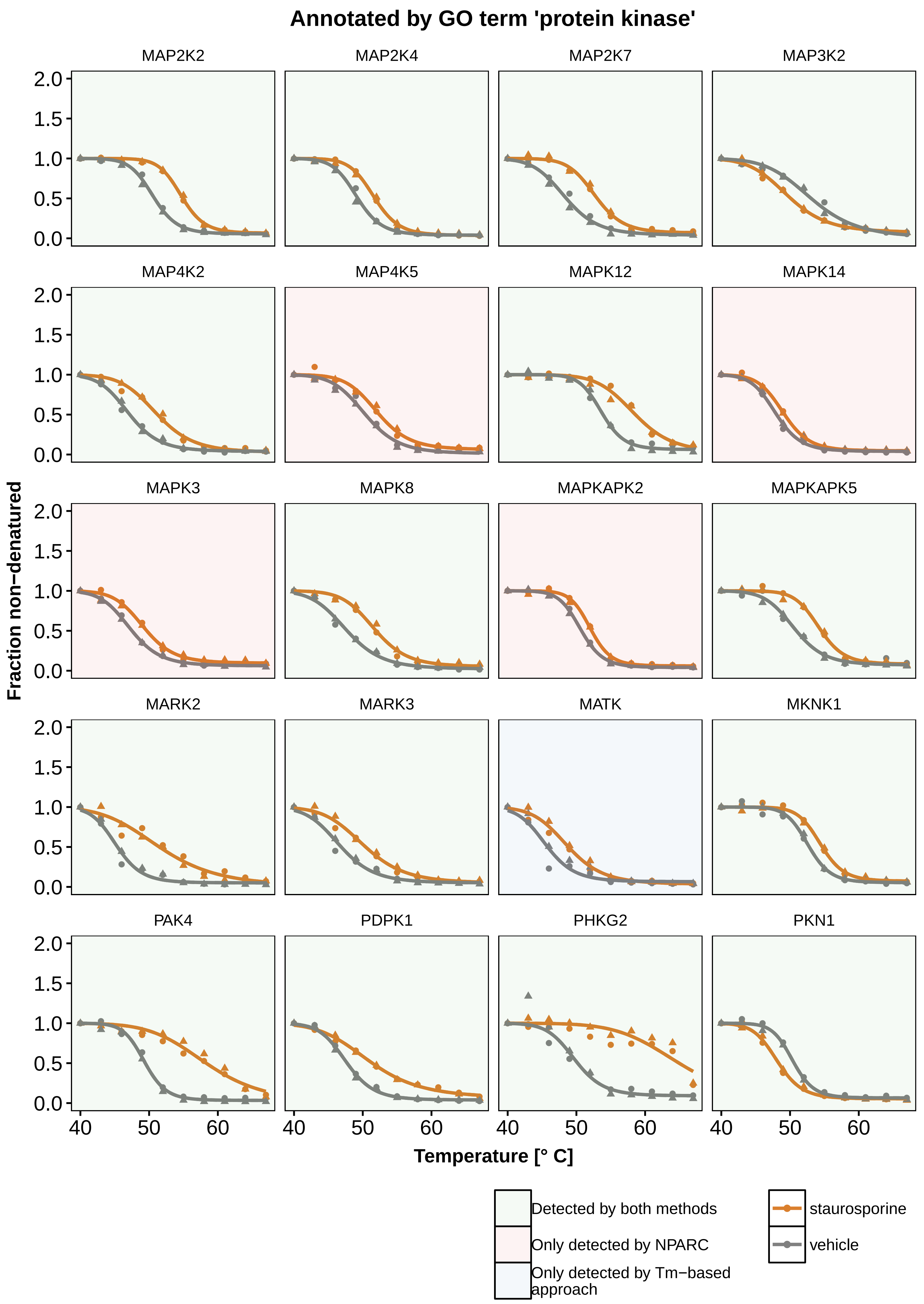
Annotated by GO term 'protein kinase'
MAP2K2
MAP2K4
MAP2K7
MAP3K2
MAP4K2
MAP4K5
MAPK12
MAPK14
MAPK3
MAPK8
MAPKAPK2
MAPKAPK5
MARK2
MARK3
MATK
MKNK1
PAK4
PDPK1
PHKG2
PKN1
Fraction non−denatured
Temperature [° C]
Detected by both methods
Only detected by NPARC
Only detected by Tm−based approach
staurosporine
vehicle

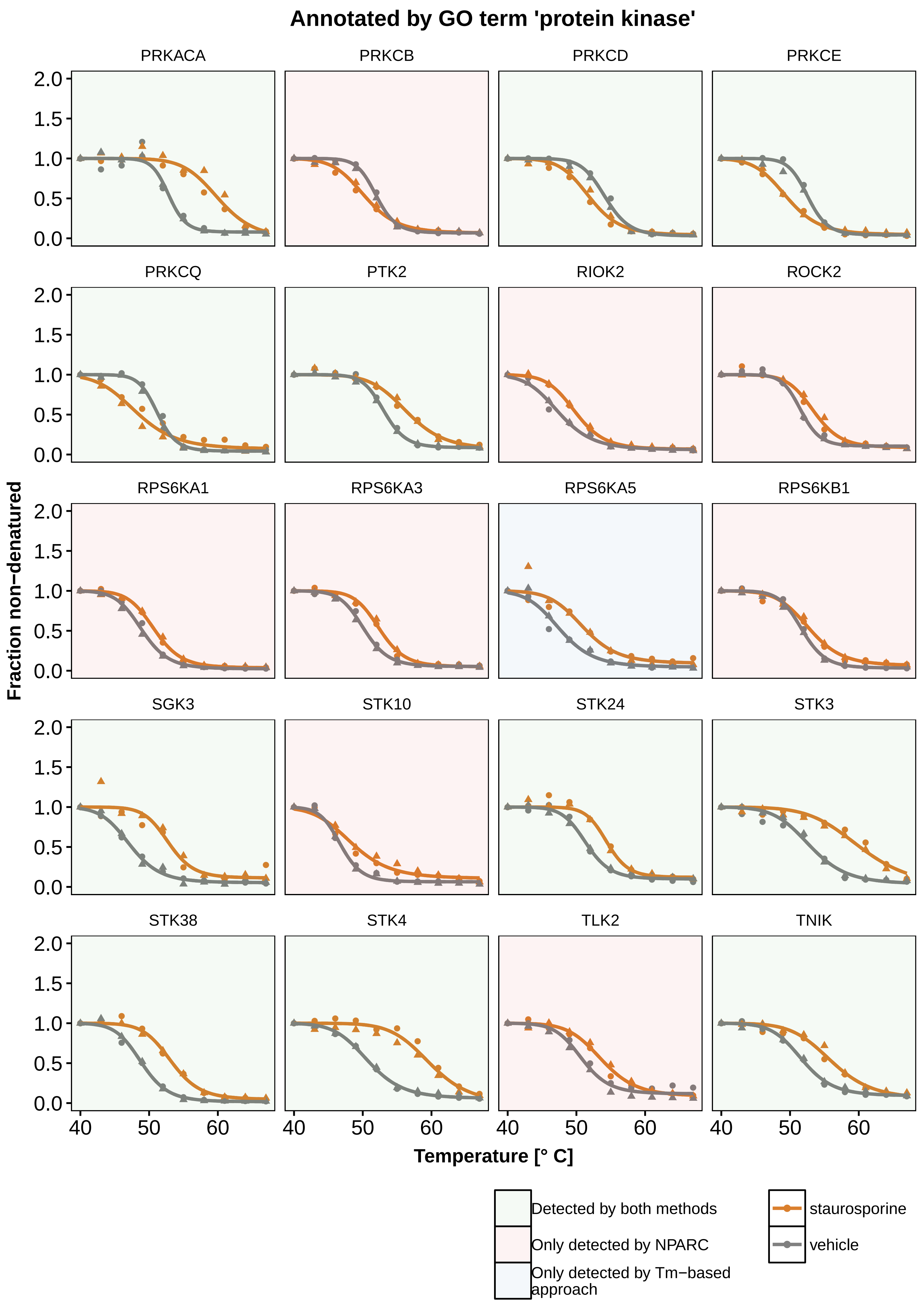
Annotated by GO term 'protein kinase'
PRKACA
PRKCB
PRKCD
PRKCE
PRKCQ
PTK2
RIOK2
ROCK2
RPS6KA1
RPS6KA3
RPS6KA5
RPS6KB1
SGK3
STK10
STK24
STK3
STK38
STK4
TLK2
TNIK
Fraction non−denatured
0.0
0.5
1.0
1.5
2.0
40
50
60
Temperature [° C]
Detected by both methods
Only detected by NPARC
Only detected by Tm−based approach
staurosporine
vehicle

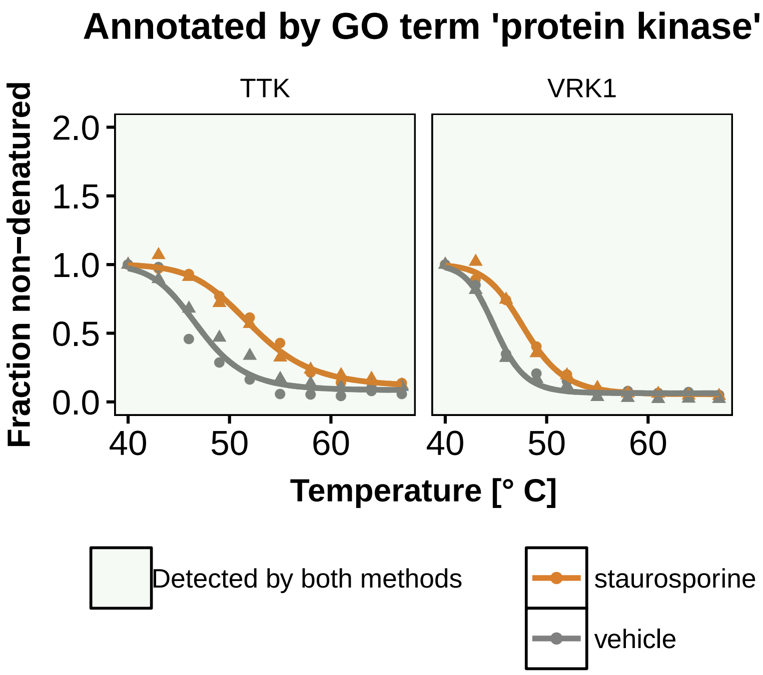
Annotated by GO term 'protein kinase'
TTK
VRK1
Fraction non−denatured
2.0
1.5
1.0
0.5
0.0
40
50
60
40
50
60
Temperature [° C]
Detected by both methods
staurosporine
vehicle

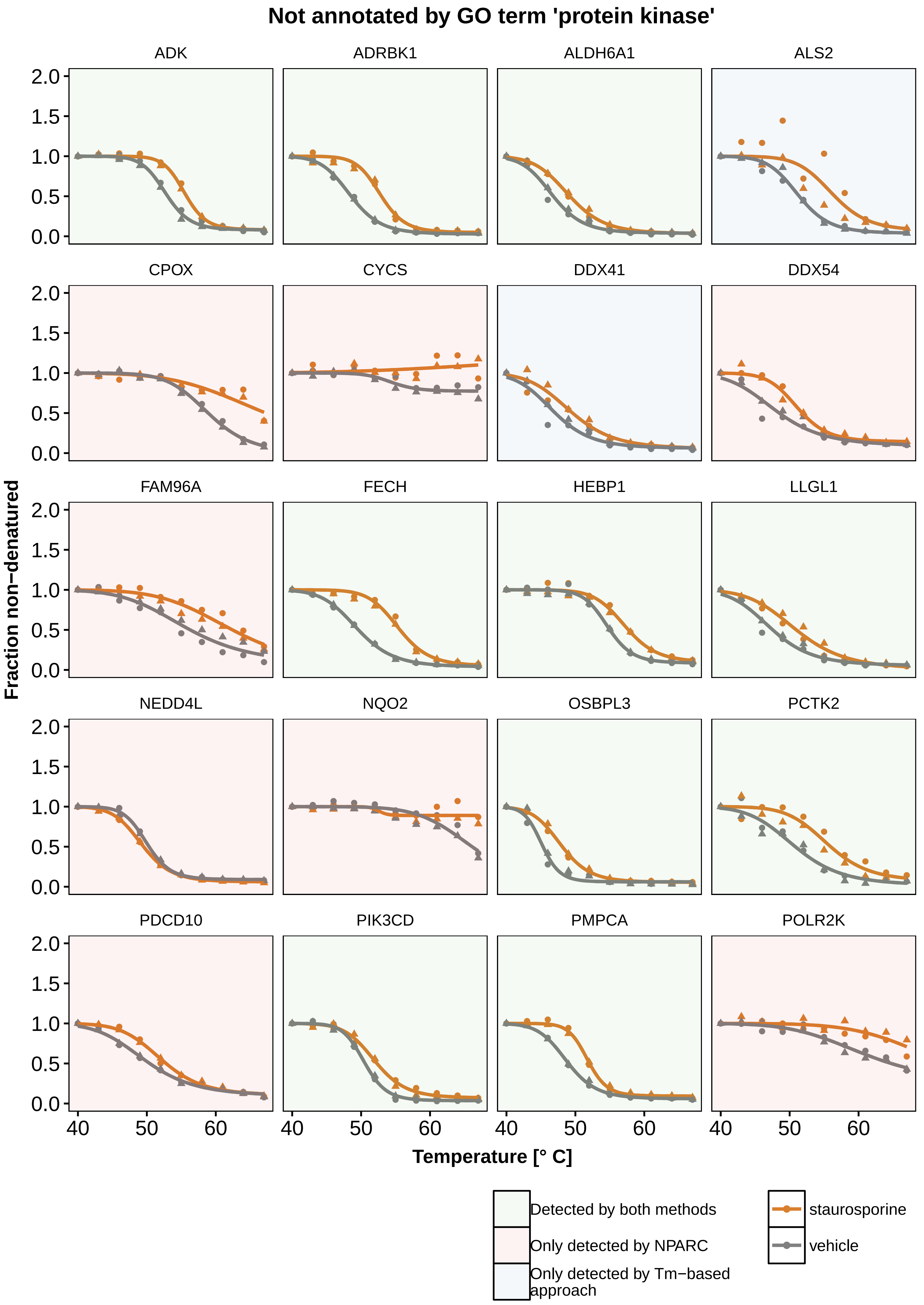
Not annotated by GO term 'protein kinase'
ADK
ADRBK1
ALDH6A1
ALS2
CPOX
CYCS
DDX41
DDX54
FAM96A
FECH
HEBP1
LLGL1
NEDD4L
NQO2
OSBPL3
PCTK2
PDCD10
PIK3CD
PMPCA
POLR2K
Fraction non−denatured
0.0
0.5
1.0
1.5
2.0
40
50
60
Temperature [° C]
Detected by both methods
Only detected by NPARC
Only detected by Tm−based approach
staurosporine
vehicle

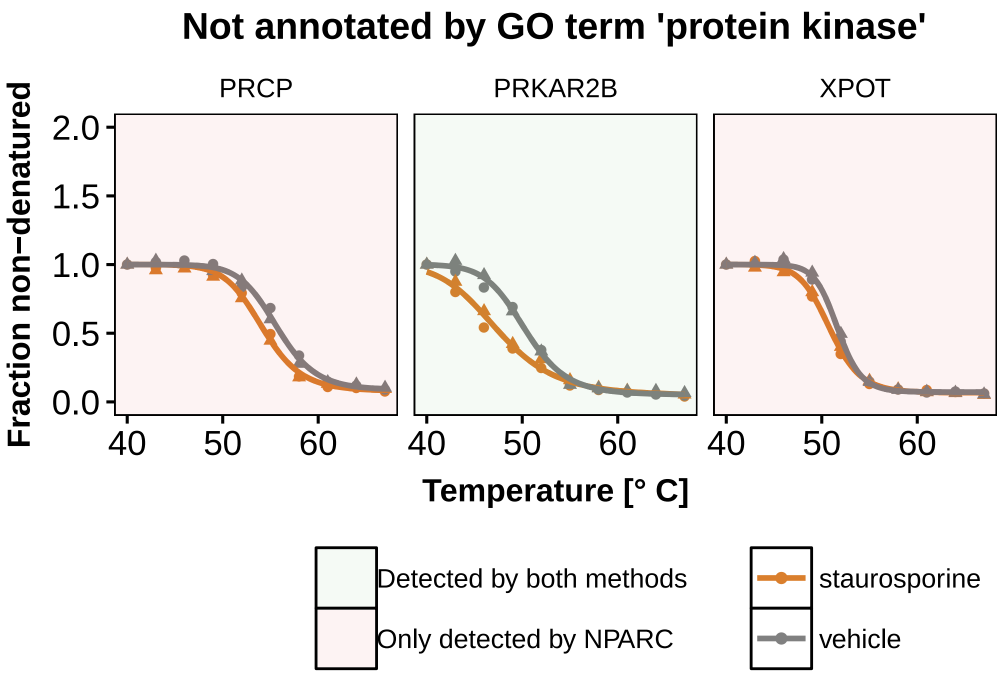
Not annotated by GO term 'protein kinase'
PRCP
PRKAR2B
XPOT
Fraction non−denatured
2.0
1.5
1.0
0.5
0.0
40
50
60
Temperature [° C]
Detected by both methods
Only detected by NPARC
staurosporine
vehicle
