# Supplementary material for: Nonparametric Analysis of Thermal Proteome Profiles Reveals Novel Drug-binding Proteins
Source: Mol Cell Proteomics. 2019 Oct 3;18(12):2506–15. doi: 10.1074/mcp.TIR119.001481 (PMC6885700; doi:10.1074/mcp.TIR119.001481)
Supplement: Supplementary Figure S7 [file 144658_1_supp_388215_px6cr4.pdf]

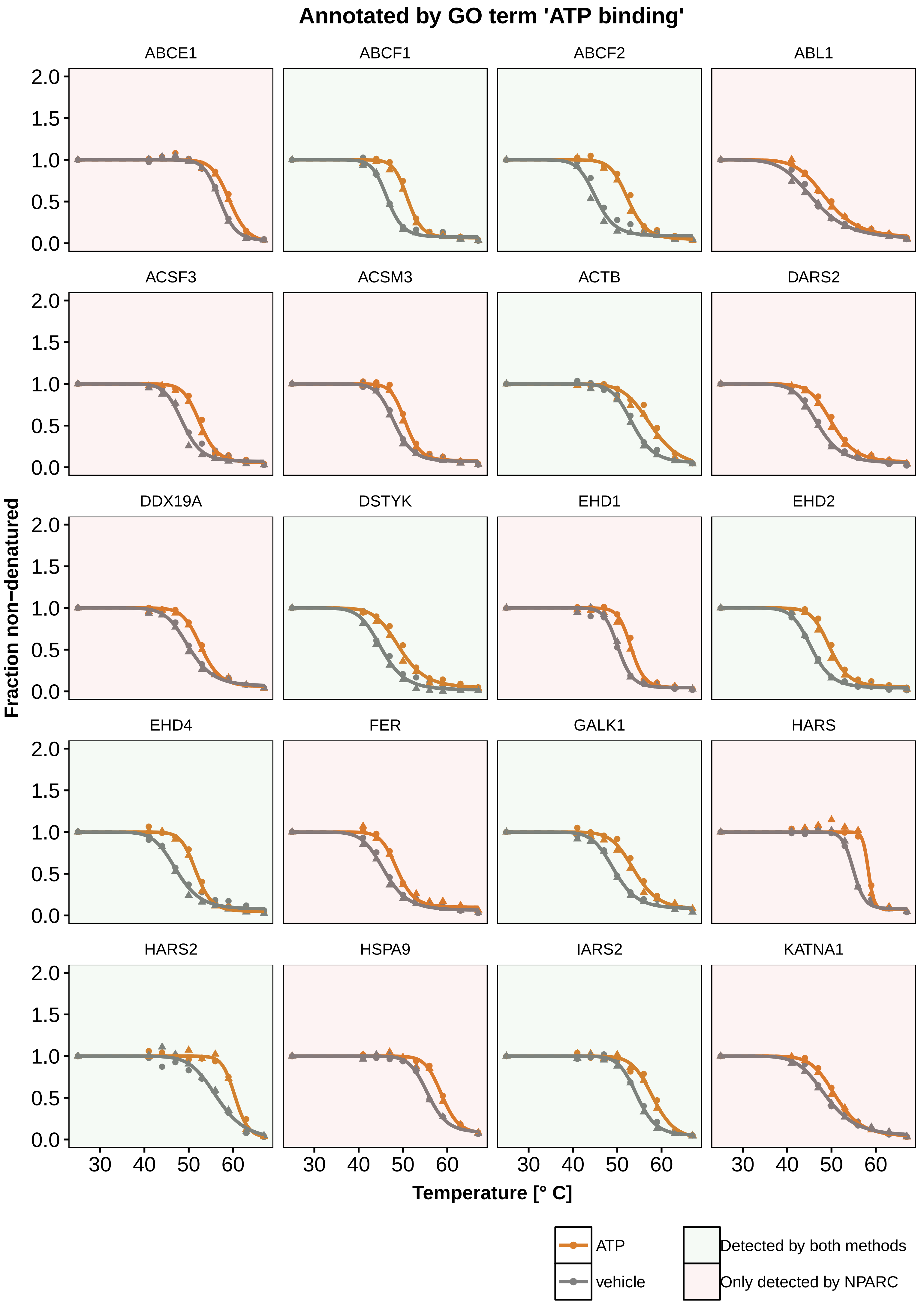

Annotated by GO term 'ATP binding'
ABCE1
ABCF1
ABCF2
ABL1
ACSF3
ACSM3
ACTB
DARS2
DDX19A
DSTYK
EHD1
EHD2
EHD4
FER
GALK1
HARS
HARS2
HSPA9
IARS2
KATNA1
Fraction non−denatured
0.0
0.5
1.0
1.5
2.0
30
40
50
60
Temperature [° C]
ATP
vehicle
Detected by both methods
Only detected by NPARC

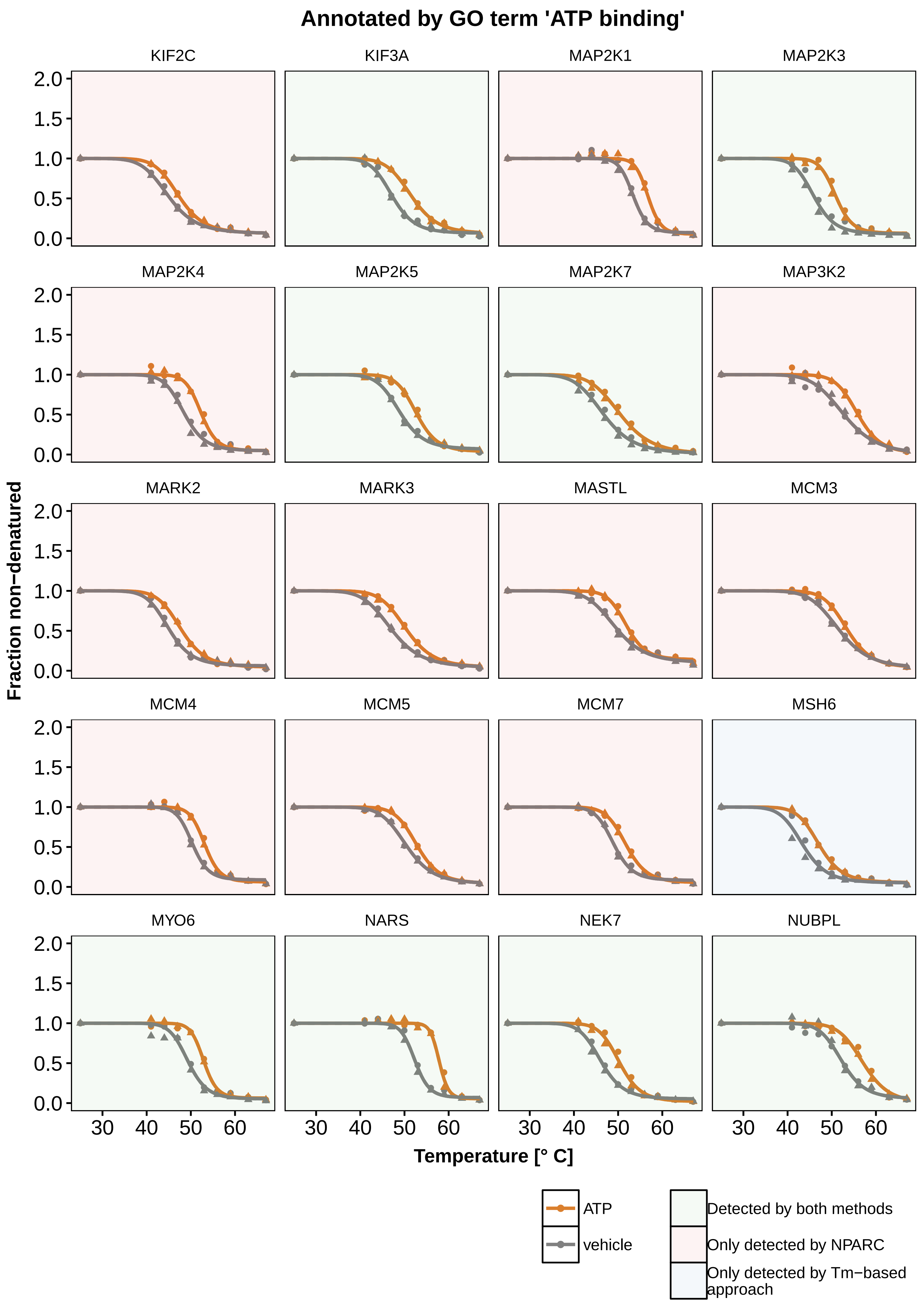
Annotated by GO term 'ATP binding'
KIF2C
KIF3A
MAP2K1
MAP2K3
MAP2K4
MAP2K5
MAP2K7
MAP3K2
MARK2
MARK3
MASTL
MCM3
MCM4
MCM5
MCM7
MSH6
MYO6
NARS
NEK7
NUBPL
Fraction non−denatured
Temperature [° C]
0.0
0.5
1.0
1.5
2.0
30
40
50
60
ATP
vehicle
Detected by both methods
Only detected by NPARC
Only detected by Tm−based approach

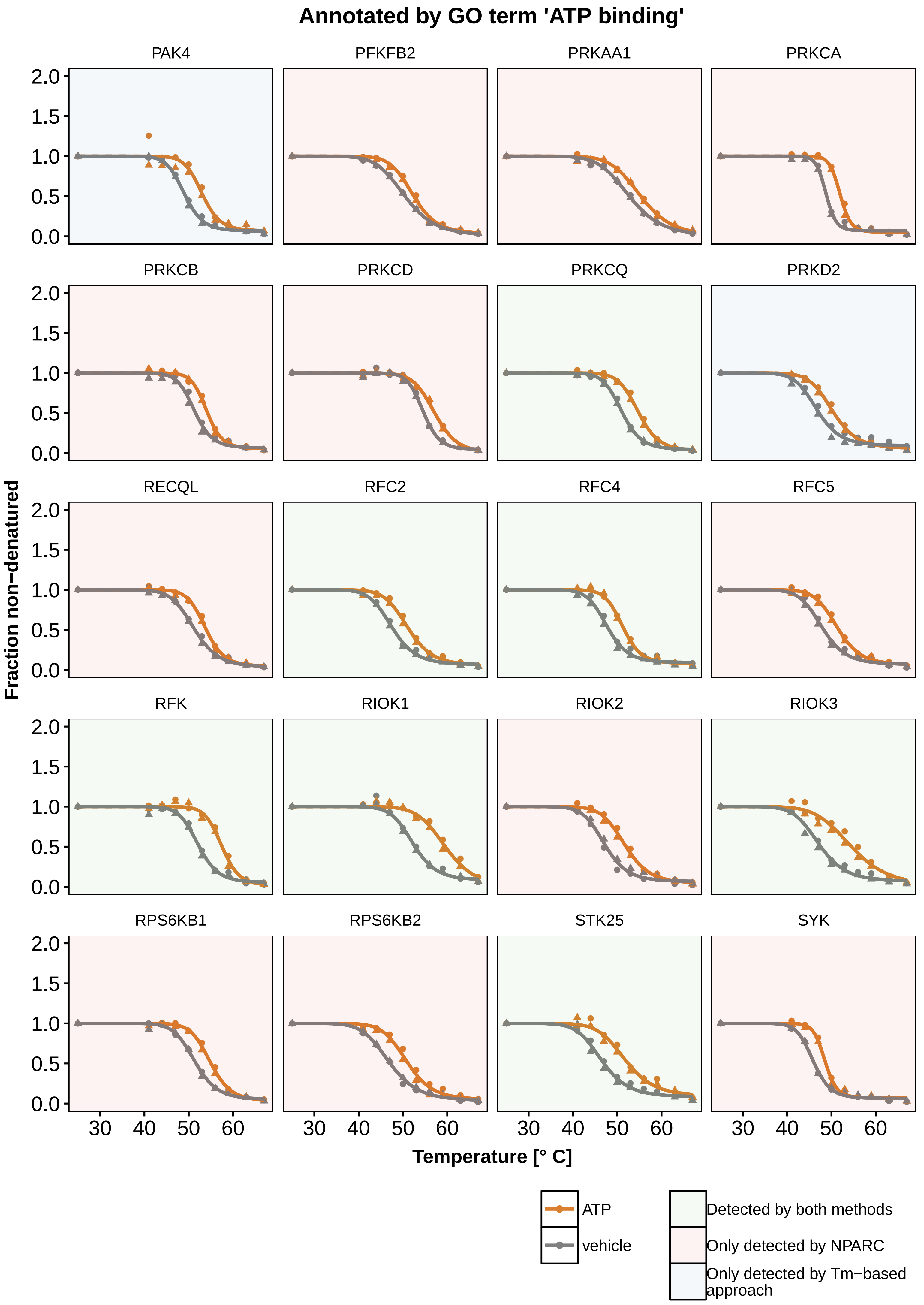

Annotated by GO term 'ATP binding'
PAK4
PFKFB2
PRKAA1
PRKCA
PRKCB
PRKCD
PRKCQ
PRKD2
RECQL
RFC2
RFC4
RFC5
RFK
RIOK1
RIOK2
RIOK3
RPS6KB1
RPS6KB2
STK25
SYK
Fraction non−denatured
2.0
1.5
1.0
0.5
0.0
30
40
50
60
Temperature [° C]
ATP
vehicle
Detected by both methods
Only detected by NPARC
Only detected by Tm−based approach

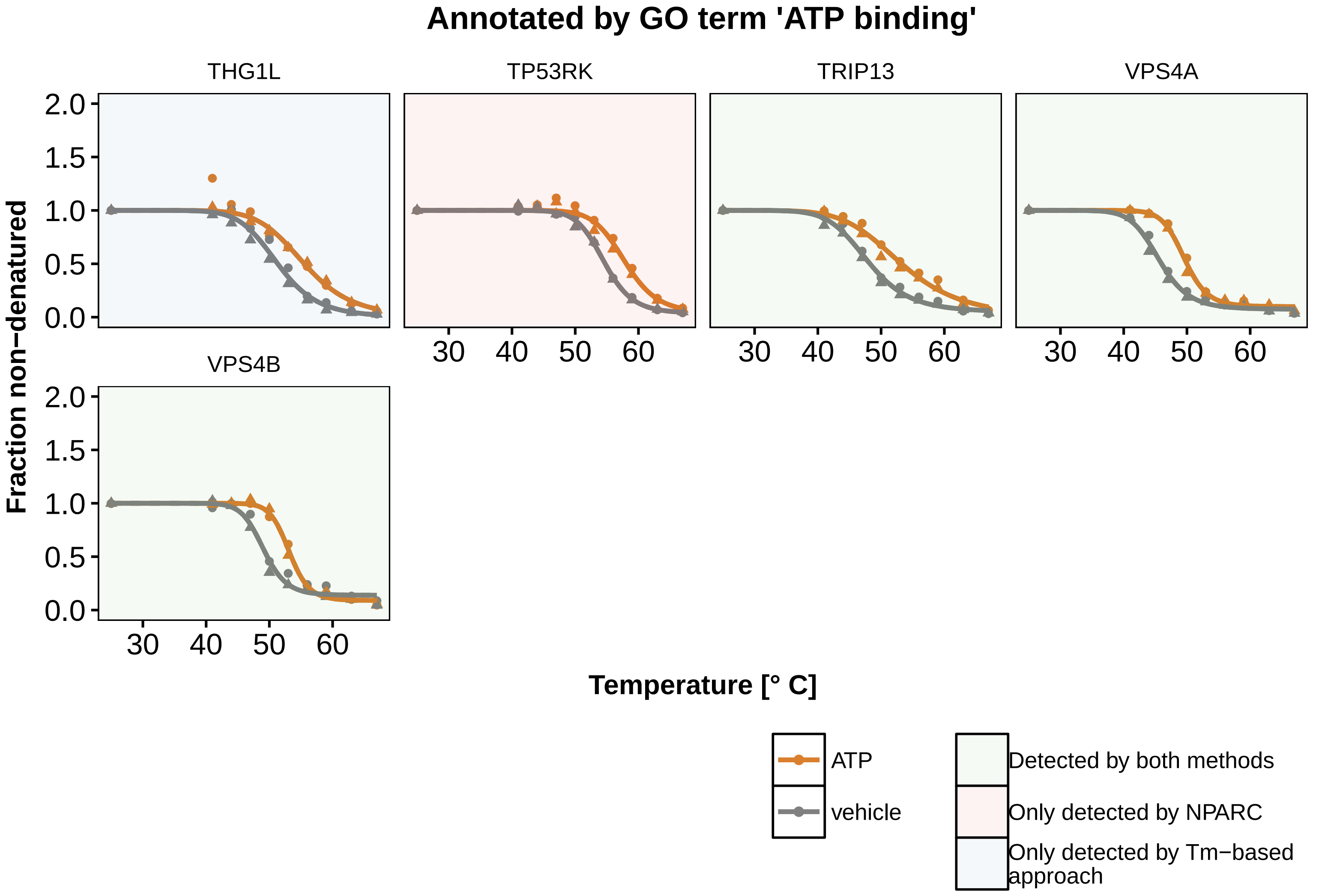
Annotated by GO term 'ATP binding'
THG1L
TP53RK
TRIP13
VPS4A
VPS4B
Fraction non−denatured
Temperature [° C]
ATP
vehicle
Detected by both methods
Only detected by NPARC
Only detected by Tm−based approach

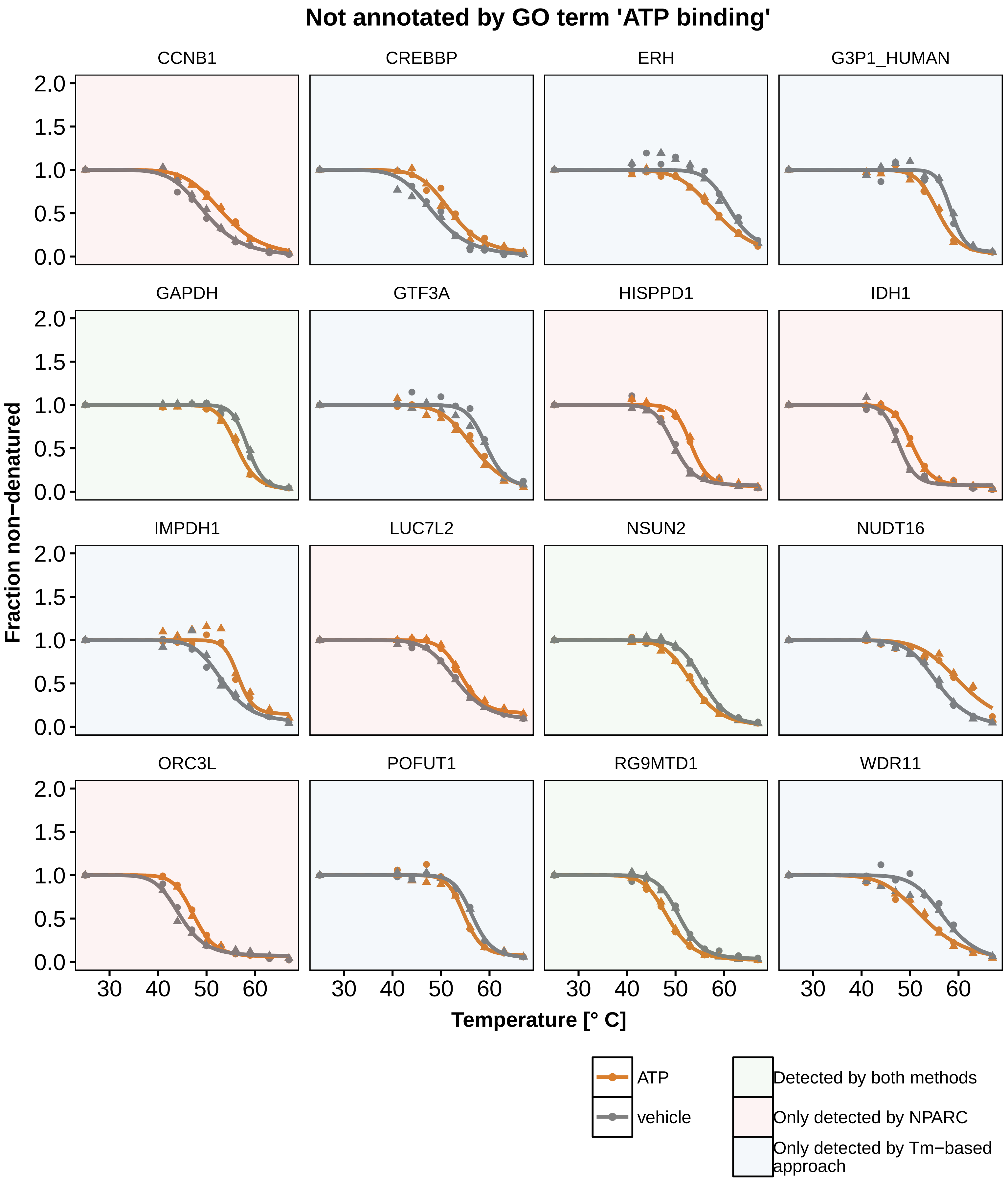
Not annotated by GO term 'ATP binding'
CCNB1
CREBBP
ERH
G3P1_HUMAN
GAPDH
GTF3A
HISPPD1
IDH1
IMPDH1
LUC7L2
NSUN2
NUDT16
ORC3L
POFUT1
RG9MTD1
WDR11
Fraction non−denatured
0.0
0.5
1.0
1.5
2.0
30
40
50
60
Temperature [° C]
ATP
vehicle
Detected by both methods
Only detected by NPARC
Only detected by Tm−based approach
